# Novel Automated Chemiluminescent Immunoassay for the Detection of Autoantibodies Against Aquaporin-4 in Neuromyelitis Optica Spectrum Disorders

**DOI:** 10.3390/diagnostics15030298

**Published:** 2025-01-27

**Authors:** Nozomi Yamazaki, Toshiyuki Takahashi, Tatsuro Misu, Yukihiro Nishikawa

**Affiliations:** 1 Medical & Biological Laboratories Co., Ltd., Tokyo 105-0012, Japan; 2 Department of Neurology, Tohoku University Graduate School of Medicine, Sendai 980-8574, Japan; 3 Department of Neurology, National Hospital Organization Yonezawa National Hospital, Yonezawa 992-1202, Japan

**Keywords:** aquaporin-4, aquaporin-4 antibodies, automated, cell-based assays, chemiluminescent, immunoassay, neuromyelitis optica spectrum disorders, NMO-IgG

## Abstract

**Background/Objectives**: Neuromyelitis optica spectrum disorder (NMOSD) is an autoimmune-related neurological disease that primarily affects the optic nerve and spinal cord. According to current international consensus guidelines for NMOSD, confirming the presence of aquaporin-4 immunoglobulin G antibody (AQP4-IgG) is one of the most important diagnostic criteria because AQP4-IgG is a significant diagnostic biomarker of NMOSD. Several assays are currently available for detecting AQP4-IgG, including cell-based assays (CBAs) and enzyme-linked immunosorbent assays (ELISAs). However, each assay has certain disadvantages, including insufficient sensitivity and specificity, the need for sophisticated techniques, and semi-quantitative results. **Methods**: We developed a fully automated chemiluminescent enzyme immunoassay (CLEIA) to detect AQP4-IgG (AQP4-CLEIA). This assay utilizes the recombinant antigen purified from the newly generated AQP4-M23 stably expressing Chinese hamster ovary cell line and an anti-AQP4 monoclonal antibody as a calibrator. **Results**: In analytical performance studies, the assay demonstrates good precision and linearity over the entire measurement range. Moreover, this assay showed no high-dose hook effect and interference from endogenous substances. In clinical validation studies, patients with AQP4-IgG positive NMOSD, multiple sclerosis, or myelin oligodendrocyte glycoprotein antibody-associated disorder and healthy individuals were tested. A cutoff value of 10.0 U/mL was determined by receiver operating characteristic curves based on the results of a microscopic live CBA. The sensitivity and specificity for AQP4-IgG-positive NMOSD were 97.0% and 100.0%, respectively, at the cutoff value. **Conclusions**: The results suggest that AQP4-CLEIA is a convenient automated method for measuring AQP4-IgG titers in hospitals and clinical laboratories, offering an effective alternative to the gold-standard CBA.

## 1. Introduction

Neuromyelitis optica spectrum disorder (NMOSD) is an autoimmune-related disease of the central nervous system (CNS), which involves demyelination and neurological deficits of the optic nerve, spinal cord, and brainstem [1]. It can result in a variety of devastating sequelae, including permanent blindness, paralysis, and even death [1,2]. Moreover, patients with NMOSD frequently experience severe and persistent neuropathic pain, which significantly impacts quality of life [3]. This autoimmune disease is characterized by the presence of aquaporin-4 immunoglobulin G antibodies (AQP4-IgG) in the patient’s serum [1].

According to current international consensus guidelines for NMOSD, confirming the presence of AQP4-IgG in the pathophysiology of NMOSD is a key diagnostic criterion [1]. AQP4-IgG is highly useful for differentiating NMOSD from other inflammatory diseases of the CNS, such as multiple sclerosis (MS) and myelin oligodendrocyte glycoprotein antibody-associated disease (MOGAD) [1,4,5]. Although these neurological diseases share some clinical features, their immunopathogenesis differs and they require appropriate clinical treatments [1,4]. Previous studies have reported that treatment of patients with NMOSD with immunomodulating agents used for MS, such as interferon beta and glatiramer acetate, may be harmful, with an increased probability of relapse and the emergence of new lesions [6,7]. Therefore, specific testing for AQP4-IgG is crucial in clinical practice for properly diagnosing and treating these neurological disorders.

Several assays have been developed to detect AQP4-IgG [8]; however, currently, the International Panel for NMO Diagnostics strongly recommends cell-based assays (CBAs) using microscopy or flow cytometry [1]. Microscopic CBAs have shown a significantly higher mean diagnostic accuracy (e.g., sensitivity and specificity) than other methods [5,8,9], which is due to the retention of the native form of AQP4-IgG binding on transfected mammalian cells expressing human AQP4. However, microscopic CBAs provide only semi-quantitative results, are observer-dependent and time-consuming, are not yet widely available, and need to be analyzed at a specialized laboratory [5,8]. While a commonly used commercial enzyme-linked immunosorbent assay (ELISA) kit (AQP4-ELISA) can complete measurements in approximately 3 h and provides observer-independent quantitative data, this kit is approximately 15% less sensitive than a microscopic CBA [9].

Recently, rapid access to test results has become crucial in clinical practice because early diagnosis and immediate immunotherapy can be life-saving for autoimmune diseases. Therefore, diagnostic laboratories are increasingly shifting to using fully automated random access systems with a focus on bead-based chemiluminescence technology [10]. The chemiluminescent enzyme immunoassay (CLEIA) is a well-established method that measures chemiluminescence to detect antigen–antibody reactions. Using antigen- or antibody-bound magnetic particles, autoantibodies or antigens in samples can be easily measured. Multiple samples can be analyzed in a short time [10], and generally, higher sensitivity than ELISA can be achieved.

To resolve problems in AQP4-IgG testing, we have developed a novel, fully automated assay with high sensitivity and specificity for the detection of AQP4-IgG by adopting the principle of the CLEIA (AQP4-CLEIA). This assay utilizes the recombinant antigen purified from the newly generated AQP4-M23 stably expressing Chinese hamster ovary cell line and an anti-AQP4 monoclonal antibody as a calibrator. We aimed to evaluate the analytical performance of this assay by measuring the AQP4-IgG titers in patient serum. As a secondary aim, we sought to establish cutoff values by comparing the AQP4-IgG titers measured using AQP4-CLEIA from patients with AQP4-IgG-positive NMOSD, MS, and MOGAD and from healthy individuals, and we compared the clinical performance of this assay with that of a microscopic live CBA.

## 2. Materials and Methods

### 2.1. Patients

Ninety-three serum samples of 33 patients with AQP4-IgG-positive NMOSD, 30 with MOGAD, and 30 with MS were obtained from Tohoku University Hospital between May 2013 and March 2023. Patients with AQP4-IgG-positive NMOSD fulfilled the 2015 diagnostic criteria [1], patients with MS fulfilled the McDonald criteria [11], and patients with MOGAD fulfilled the 2023 International MOGAD Panel proposed criteria [4]. All patients with AQP4-IgG-positive NMOSD were positive for AQP4-IgG serum as measured using a microscopic live CBA (AQP4-CBA), as described previously [12]. All patients with MOGAD were positive for MOG-IgG serum as measured using a microscopic live CBA method [13]. Ten high-titer AQP4-IgG plasma samples from patients with NMOSD were obtained from Tohoku University Hospital and used for analytical performance evaluation. One hundred twenty serum samples from apparently healthy individuals were obtained from SLR Research Corporation (Carlsbad, CA, USA). Of note, all samples were stored at −80 °C until assaying.

This study was approved by the Ethics Committee of Tohoku University Hospital (2019-1-900, 2022-1-015) and by Medical & Biological Laboratories Co., Ltd., (MBL, Tokyo, Japan; 253). Written informed consent was obtained from all patients involved in the study. The study adhered to the principles of the Declaration of Helsinki.

### 2.2. Generation of AQP4-M23 Stably Expressing Chinese Hamster Ovary Cell Line and Purification of AQP4-M23

**Figure 1:**
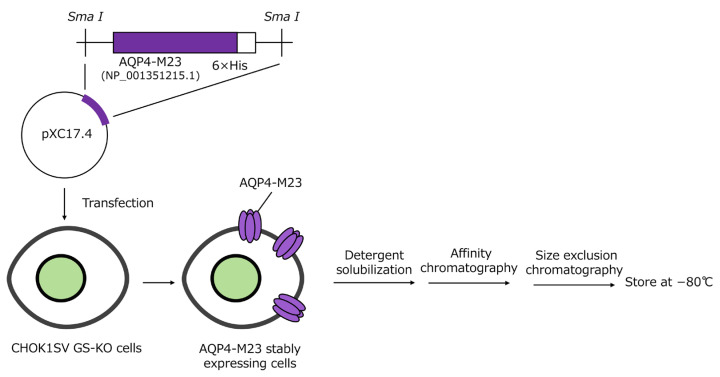
Schematic representation of AQP4-M23 expression and purification.

A full-length human AQP4-M23 ORF with a C-terminal 6× His tag, followed by a single stop-codon sequence, was synthesized and cloned into pXC17.4 (Lonza Inc., Basel, Switzerland) at the *Sma* I site. Recombinant human AQP4-M23 with a C-terminal His tag was expressed in CHOK1SV GS-KO cells using the GS Xceed™ Gene Expression System (Lonza) according to the instruction manual. AQP4-M23 expression was confirmed through Western blotting, and a stable cell clone was obtained. Cultured cells were harvested, homogenized, and purified as previously described, with some modifications [14,15]. Briefly, the cells were homogenized, and the resulting pellet was washed. The washed membranes were solubilized with 4% n-octyl-β, D-glucopyranoside. The supernatant was collected and loaded onto a HiTrap^®^ TALON^®^ crude column (Cytiva, Marlborough, MA, USA) using an AKTA pure 25 M2 system. Fractions containing AQP4-M23, as verified by sodium dodecyl sulfate-polyacrylamide electrophoresis (SDS-PAGE), were pooled and loaded onto a HiLoad 26/600 Superdex 200 pg column (Cytiva) according to the manufacturer’s instructions. Purified AQP4-M23 was assessed using SDS-PAGE and Western blotting, then quantified using the Quick Start™ Bradford protein assay (Bio-Rad Laboratories, Hercules, CA, USA) and stored at −80 °C until use. A schematic representation of AQP4-M23 expression and purification is shown in Figure 1.

### 2.3. Generation of Anti-AQP4 Monoclonal Antibody-Stable Expression Chinese Hamster Ovary Cell Line and Purification of Anti-AQP4 Monoclonal Antibody

The cDNA encoding the anti-AQP4 monoclonal antibody (AQP4-mAb) was synthesized according to a previous report [16] with some modifications and expressed using the GS Xceed™ Gene Expression System (Lonza) in CHOK1SV GS-KO cells according to the instruction manual. Cell clones were isolated by limiting dilution, and a single-clone cell line was established. Antibodies were purified using Protein A Sepharose Fast Flow (Cytiva). Fractions containing the antibodies were dialyzed against 50% (*v*/*v*) glycerol/phosphate-buffered saline. Purified antibodies were checked by SDS-PAGE and were quantified using a Nanodrop instrument (Thermo Fisher Scientific, Waltham, MA, USA). They were stored below −20 °C until use.

### 2.4. AQP4-CLEIA

The AQP4-CLEIA is a two-step assay. The AQP4-IgG analyte reacted with the paramagnetic microparticles coated with AQP4-M23 antigens. The analyte–microparticle complex was detected using alkaline phosphatase (ALP)-labeled anti-human IgG polyclonal antibodies. The reaction mixture was then exposed to a chemiluminescent substrate for luminescence in proportion to the amount of AQP4-IgG.

#### 2.4.1. AQP4-CLEIA Assay Protocol

The following procedures were performed automatically using the STACIA system (PHC Holdings Corp., Tokyo, Japan). The measurements were completed within 20 min with a maximum throughput of 270 tests/h.

**Figure 2:**
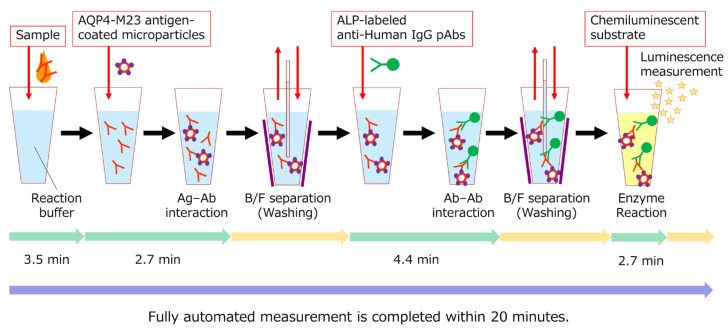
Schematic representation of AQP4-IgG measurement using a two-step method. Ab, antibody; Ag, antigen; ALP, alkaline phosphatase; pAbs, polyclonal antibodies.

In the first step, 100 μL of reaction buffer and 10 μL of the sample were mixed and incubated for approximately 3.5 min. Next, 50 μL of AQP4-M23 antigen-coated microparticles were added and incubated for approximately 2.7 min to form an analyte–microparticle complex. After the bound and free fractions were separated (B/F separation), 100 μL of ALP-labeled anti-human IgG polyclonal antibody solution was added to the analyte–microparticle complex, and the sample was incubated for approximately 4.4 min. B/F separation was then performed again, the samples were incubated with 100 μL of chemiluminescent substrate solution (CDP-Star; Applied BioSystems, Bedford, MA, USA) for 2.7 min, and the luminescence was measured using a luminometer in the STACIA CLEIA system. A schematic representation of AQP4-IgG measurements is shown in Figure 2.

#### 2.4.2. Calibrators for AQP4-CLEIA

The AQP4-mAb was used as a calibrator for the AQP4 CLEIA assay. The concentration was quantified by measuring the absorbance at 280 nm using a UV-1900 spectrophotometer (Shimadzu UV-1900; Shimadzu, Kyoto, Japan). Because no international standard for AQP4-IgG is available, we defined 1.2 ng/mL of the AQP4-mAb as 1.0 U/mL and established a quantification method for CLEIA. The calibrator concentration ranged from 0 to 500.0 U/mL (0, 7.5, 29.2, 104.2, and 500.0 U/mL) with an effective measurement range of 4.6–500.0 U/mL.

### 2.5. Evaluation Methods for AQP4-CLEIA

#### 2.5.1. Assay Precision

The precision of the assay was evaluated using intra- and inter-assay methods. To assess intra-assay precision, serum samples at three AQP4-IgG titers were analyzed six times in replicates using three distinct reagent lots. Similarly, inter-assay precision was evaluated twice per day for 3 days using three titers of AQP4-IgG samples and three reagent lots. Intra- and inter-assay validations were performed using samples with different AQP4-IgG titers.

#### 2.5.2. Limits of Quantification

The limit of quantification (LoQ) was determined in accordance with the Clinical and Laboratory Standards Institute (CLSI) protocol EP17-A2. Briefly, five low-concentration samples were prepared, and the expected values were set using two STACIA instruments and three reagent lots. Five samples were measured for 3 days in two reagent lots. The percentage of Westgard’s total error (TE%) [17] was calculated, and the LoQ was established as the lowest concentration that showed a TE% of <20%.

#### 2.5.3. Dilution Linearity

Dilution linearity was determined according to the CLSI protocol EP06-A2. The three native AQP4-IgG serum samples were serially diluted with normal serum samples. The diluted samples were tested in triplicates using a single reagent lot. The observed values were then compared with the expected values based on the corresponding concentrations of the undiluted specimens using a weighted linear regression analysis. The allowable deviation from linearity was determined to be ±15%.

#### 2.5.4. Hook Effect

The absence of a high-dose hook effect was confirmed through testing whether signal suppression occurred at analyte levels exceeding the concentration levels of the assay calibration. This was performed using a high-titer sample of approximately 3100 U/mL, which exceeded the dynamic range of the assay (500.0 U/mL).

#### 2.5.5. Influence of Endogenous Substances

The influence of endogenous substances was evaluated according to the CLSI protocol EP07-A2. Each endogenous substance was checked using Interference Check A Plus, Interference Check RF Plus (Sysmex, Kobe, Japan) and Intralipid (Sigma-Aldrich, St. Louis, MO, USA). Samples with endogenous substances and blank samples with control solutions were prepared at concentrations above those recommended in the CLSI protocol EP037-ED1. The chyle material and rheumatoid factor are not listed in the CLSI protocol EP037-ED1; thus, the manufacturer’s instructions were followed. Serum samples with three titers of AQP4-IgG were mixed with free bilirubin, conjugated bilirubin, hemoglobin, chyle material, rheumatoid factor, and triglyceride. The mean AQP4-IgG titer of each test sample was compared with the mean value of the corresponding control sample. Samples with concentration differences within ±20% were considered free of interference.

### 2.6. Statistical Analyses

In clinical validation studies, sensitivity was calculated in 33 patients with AQP4-IgG-positive NMOSD, and specificity was calculated in controls. The Wilcoxon–Mann–Whitney test was used to compare patients with AQP4-IgG-positive NMOSD and other groups. *p* values < 0.05 were considered statistically significant. Receiver operating characteristic (ROC) curve analysis and all statistical analyses were performed using Analyse-it version 6.15 (Analyse-it Software Ltd., Leeds, UK).

## 3. Results

### 3.1. Analytical Performance of AQP4-CLEIA

The precision data for the assay are shown in Table 1. The intra- and inter-assay precisions ranged from 2.5% to 6.2% and from 4.9% to 7.7%, respectively.

In accordance with CLSI protocol EP17-A2, the LoQ was defined as the concentration at which the TE% in the Westgard model was <20%. The TE% for each sample is shown in Table 2. The LoQ of the first reagent lot was 4.22 U/mL, and that of the second reagent lot was 4.54 U/mL. The reagent was validated to the first decimal place, and the final LoQ was determined to be 4.6 U/mL.

**Figure 3:**
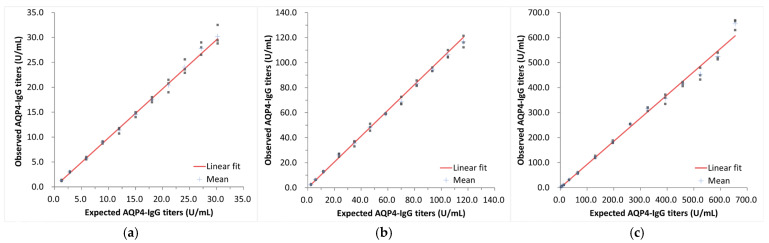
Evaluation of dilution linearity. Relationship between expected AQP4-IgG titers and observed AQP4-IgG titers with low- (**a**), middle- (**b**), and high- (**c**) titer serum samples.

Dilution linearity studies were conducted in accordance with CLSI protocol EP06-A2. The dilution linearity of 3.0–524.0 U/mL was confirmed through testing in three specimens (Figure 3a–c).

**Figure 4:**
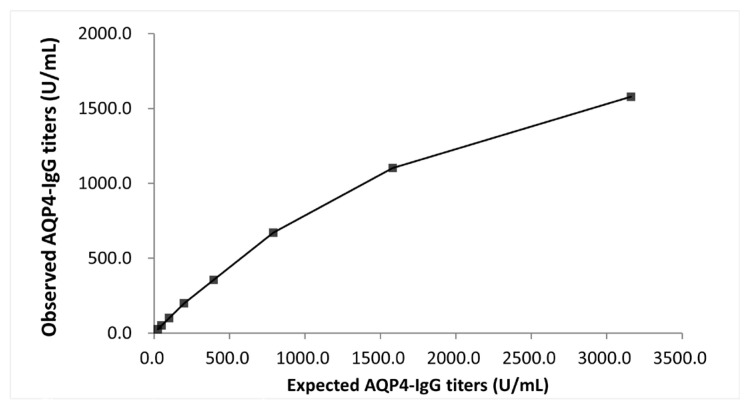
Evaluation of the hook effect. The relationship between expected AQP4-IgG titers and observed AQP4-IgG titers beyond the dynamic range.

The hook effect was evaluated in a serum sample with high AQP4-IgG titers. No high-dose hook effect was observed under the testing conditions up to approximately 3100 U/mL (Figure 4). Based on these findings, the dynamic range of the reagent was defined as 4.6–500.0 U/mL.

The effects of the endogenous substances are presented in Table 3. The measurement value differences between the test and control samples ranged from a minimum of −2.8% to 5.6%, and the 95% confidence intervals included zero. Thus, no significant difference was observed. The concentrations of the endogenous substances did not affect the AQP4-IgG titers listed in Table 3.

### 3.2. Clinical Validation in the Study Participants

**Figure 5:**
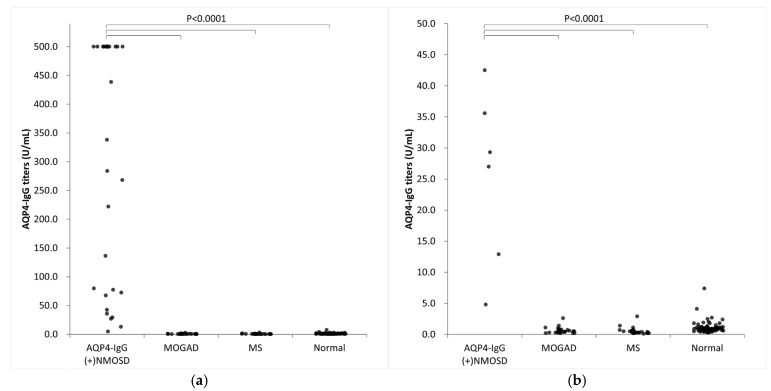
Clinical validation of AQP4-CLEIA. AQP4-IgG titers in 33 patients with AQP4-IgG-positive NMOSD, 30 with MOGAD, and 30 with MS, and in 120 healthy controls. (**a**) Overview, (**b**) enlarged view. AQP4-IgG (+) NMOSD, AQP4-IgG-positive neuromyelitis optica spectrum disorder; normal, healthy controls; MOGAD, myelin oligodendrocyte glycoprotein antibody-associated disease; MS, multiple sclerosis.

**Figure 6:**
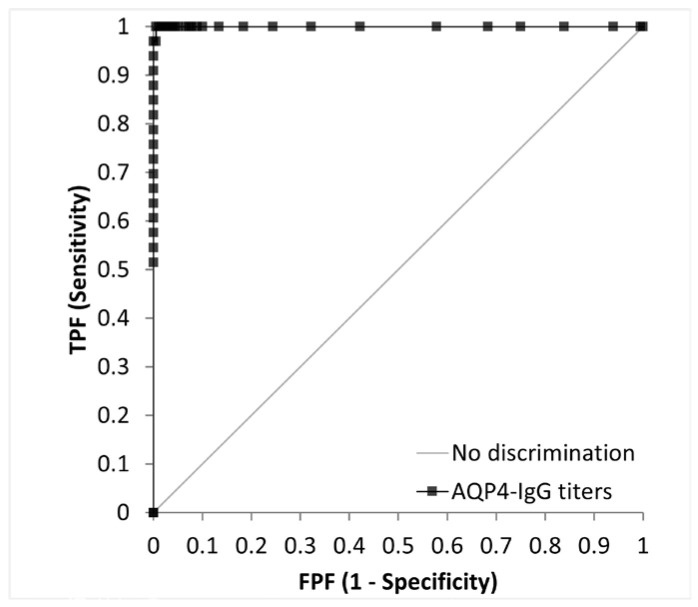
Receiver operating characteristic curve analysis of AQP4-IgG titers determined by AQP4-CLEIA in 33 patients with AQP4-IgG-positive NMOSD, 30 with MOGAD, and 30 with MS, and in 120 healthy controls. MOGAD, myelin oligodendrocyte glycoprotein antibody-associated disease; MS, multiple sclerosis; NMOSD, neuromyelitis optica spectrum disorder.

We evaluated the AQP4-IgG titers in 33 patients with AQP4-IgG-positive NMOSD, 30 with MOGAD, and 30 with MS, and in 120 healthy controls (Figure 5a,b). AQP4-CLEIA measurements were performed using three reagent lots, and the mean of the measured values was used for subsequent analyses. In patients with AQP4-IgG-positive NMOSD, AQP4-IgG titers ranged from 4.8 U/mL to ≥500.0 U/mL. Of the 33 patients with AQP4-IgG-positive NMOSD, 17 had titers above the assay range, with AQP4-CBA results as high as >4096 times the AQP4-IgG titers’ results (Table 4). Patients with MOGAD and MS had lower AQP4-IgG titers and mean values below the LoQ (<4.6 U/mL). Almost all healthy controls showed values below the LoQ (<4.6 U/mL), except for one (7.4 U/mL). A cutoff value of 10.0 U/mL was determined by an ROC curve analysis (Figure 6). At this cutoff, the sensitivity was 97.0% (95% confidence interval [CI] 84.7–99.5), the specificity was 100.0% (95% CI 97.9–100.0%), and the positive percentage agreement with AQP4-CBA was 97.0%.

## 4. Discussion

The objective of this study was to develop a high-sensitivity and high-specificity AQP4-CLEIA using the STACIA system, which is commonly employed in clinical laboratories [18,19]. The system was constructed using a highly purified recombinant antigen obtained from an AQP4-M23 stably expressed Chinese hamster ovary cell line, and an anti-AQP4 monoclonal antibody was used as a calibrator. Furthermore, the system was fully automated, enabling the completion of measurements within 20 min and the processing of up to 270 tests/h.

We evaluated the analytical performance of AQP4-CLEIA using several methods. As demonstrated in previous studies, AQP4-IgG titers yield variable results, depending on the assays and facilities used [8,20]. These reports indicate a lack of standardization of AQP4-IgG measurements, which could significantly impact the results of large multicenter studies and routine testing in clinical laboratories. Measurement standardization is essential to characterize the clinical and pathological features of patients accurately. Considering the absence of referential or commercially available standards for AQP4-IgG, we utilized the AQP4-mAb as an in-house standard material to standardize the measurement. We defined 1.2 ng/mL of AQP4-mAb as 1.0 U/mL. As this is a proprietary measurement unit established using our own method, we expressed it in arbitrary units of U/mL rather than ng/mL. The AQP4-IgG titers for AQP4-CLEIA were determined using assay calibrators prepared from AQP4-mAb, which served as the in-house standard material. This methodology facilitated the traceability of AQP4-IgG titers to the concentration of the in-house standard material, thereby maintaining consistency in the measurement values. The new assay, AQP4-CLEIA, demonstrated high precision with coefficients of variation ranging 2.5–6.2% for intra-assay precision and 4.9–7.7% for inter-assay precision. Furthermore, the assay demonstrated excellent linearity over the measurement range, and no hook effect was observed up to approximately 3100 U/mL. The LoQ was established as 4.6 U/mL, with a measurement range of 4.6–500.0 U/mL. The assay demonstrated acceptable performance against endogenous substances, including hemoglobin, free bilirubin, conjugated bilirubin, chyle material, rheumatoid factor, and triglycerides. Therefore, we conclude that the assay has excellent analytical accuracy and is suitable for use in multicenter studies conducted at various facilities and for routine testing in clinical laboratories.

In the clinical validation studies, 33 patients with AQP4-IgG-positive NMOSD, 30 with MOGAD, 30 with MS, and 120 healthy controls were evaluated. We conducted an ROC curve analysis. The cutoff value of AQP4-CLEIA was determined to be 10.0 U/mL, with a sensitivity of 97.0% (95% CI: 84.7–99.5%) and a specificity of 100.0% (95% CI: 97.9–100%). In 1 of the 33 patients with AQP4-IgG-positive NMOSD, the assay yielded low titers of 4.8 U/mL. The patient also showed low titers of AQP4-CBA. In consideration of the clinical specificity, the patient with low titers tested negative via the AQP4-CLEIA assay.

As shown in Table 5, AQP4-CLEIA has some advantages over AQP4-CBA and AQP4-ELISA. The assay is a fully automated system and can be completed within 20 min, making it highly useful in clinical laboratories that necessitate rapid results. Moreover, AQP4-CLEIA has excellent sensitivity and specificity comparable with those of AQP4-CBA. A lack of sensitivity may lead to a delayed determination of an appropriate treatment strategy, requiring retesting or alternative assays. A lack of specificity may lead to incorrect diagnostic outcomes, increasing the probability of inappropriate therapeutic treatment. This aspect is particularly relevant because of the lack of established biomarkers for MS and the limited availability of antibody tests, such as those for MOGAD and anti-NMDA receptor antibody encephalitis, in certain clinical laboratories [4,11,21]. Therefore, this novel, fully automated AQP4-CLEIA has the potential to fulfill clinical laboratory requirements and is expected to become an alternative to AQP4-CBA in the future.

However, this study had some limitations. First, the control samples were limited to those from patients with MS, those with MOGAD, and healthy individuals; and samples from patients with clinically differentiated diseases were lacking. Therefore, future studies should incorporate objective validation with additional samples from patients with other neurological disorders to assess the accurate clinical performance of AQP4-CLEIA. Second, the measurement sample was limited to serum, and the efficacy of AQP4-CLEIA was not evaluated for alternative measurement samples, such as plasma or cerebrospinal fluid. As outlined in international guidelines [1] and previous studies [8,9], AQP4-IgG is typically measured in serum for NMOSD diagnosis prior to initiating acute treatment. Nevertheless, future investigations into the applicability of AQP4-CLEIA to plasma and cerebrospinal fluid may potentially yield novel clinical findings unique to this reagent. Finally, a direct comparison of AQP4-CLEIA with AQP4-ELISA was not conducted. In this study, we focused on comparing AQP4-CLEIA and a gold-standard method—AQP4-CBA—because the international guideline states that AQP4-CBA is strongly recommended for AQP4-IgG testing due to its high average diagnostic accuracy (e.g., sensitivity and specificity). Nevertheless, in Japan, AQP4-CBA is not reimbursed, and only AQP4-ELISA is currently covered by insurance in clinical practice. It is crucial to acknowledge that the units of measurement (U/mL) in AQP4-CLEIA and AQP4-ELISA are incompatible. This incompatibility arises from the differing definitions of the units of measurement (U/mL) employed in these assays and the fundamentally distinct measurement principles underlying these assays. As illustrated in Figure 2, the AQP4-CLEIA method utilizes a measurement principle analogous to that of the CBA method, which detects AQP4-IgG bound to an antigen using a fluorochrome- or ALP-labeled anti-human IgG antibody. In contrast, the AQP4-ELISA method relies on the formation of a cross-link between immobilized AQP4 and biotinylated AQP4 by anti-AQP4 antibodies (with the possibility of reacting with immunoglobulins other than AQP4-IgG [e.g., AQP4-IgM]). Subsequently, the biotinylated antigen is detected using streptavidin peroxidase and a chromogenic peroxidase substrate. This principle mirrors that of a commercially available glutamic acid decarboxylase autoantibody ELISA kit [22]. Owing to these differences, no correlation may be observed between the values obtained with AQP4-CLEIA and AQP4-ELISA. Therefore, future studies are needed to compare AQP4-CLEIA and AQP4-ELISA using measurements from the same cohort.

## 5. Conclusions

Our new AQP4-CLEIA exhibited favorable clinical performance, nearly equivalent to that of the CBA method—the gold-standard procedure for the AQP4-IgG assay. Moreover, this assay is an automated method that exhibits excellent analytical performance, offering the high precision, simplicity, and high throughput required for routine testing in hospitals and clinical laboratories. Further clinical trials are currently underway in Japan with the objective of designing more reliable studies and validating cutoff values.

## Figures and Tables

**Table 1:** Intra- and inter-assay validations.

AQP4-IgG Titers of Samples	Precision	Intra-Assay Precision			Inter-Assay Precision		
	Reagent Lot	Lot 1	Lot 2	Lot 3	Lot 1	Lot 2	Lot 3
Low	Mean (U/mL)	18.9	17.6	19.3	17.9	18.0	17.9
	SD (U/mL)	0.74	0.94	0.93	0.97	0.98	1.04
	CV	3.9%	5.3%	4.8%	5.4%	5.4%	5.8%
Middle	Mean (U/mL)	89.3	88.5	84.1	84.8	82.4	84.6
	SD (U/mL)	5.50	2.62	2.10	6.24	5.70	6.48
	CV	6.2%	3.0%	2.5%	7.4%	6.9%	7.7%
High	Mean (U/mL)	427.5	378.2	447.5	419.8	384.6	409.0
	SD (U/mL)	11.73	20.27	12.04	22.90	18.96	30.39
	CV	2.7%	5.4%	2.7%	5.5%	4.9%	7.4%

CV, coefficient of variation; SD, standard deviation.

**Table 2:** Total error% calculations for reagent Lot 1 and Lot 2.

Reagent Lot		Lot 1					Lot 2				
Sample No.		1	2	3	4	5	1	2	3	4	5
Estimated value (U/mL)		5.62	4.54	4.28	4.22	3.47	5.62	4.54	4.28	4.22	3.47
Day 1	1	5.73	4.77	4.92	4.30	3.73	5.28	4.92	4.82	4.32	3.58
	2	6.06	4.69	4.82	4.28	3.41	6.12	5.06	4.82	4.45	3.22
	3	5.94	4.51	4.54	3.70	3.73	5.91	4.81	4.97	4.29	3.46
Day 2	1	6.26	4.50	4.72	4.35	2.98	5.16	4.82	5.20	4.95	4.01
	2	6.15	4.12	4.76	4.06	3.67	5.04	4.69	5.08	4.08	3.49
	3	5.77	4.22	4.34	4.34	3.75	5.74	4.89	4.70	4.71	3.35
Day 3	1	4.75	3.94	4.42	3.75	3.14	6.02	4.55	4.48	4.20	3.38
	2	5.67	4.10	4.56	4.15	3.18	5.98	4.86	4.64	3.58	3.14
	3	5.78	4.37	4.50	4.06	2.83	5.68	4.75	4.49	4.16	2.90
Mean (U/mL)		5.79	4.36	4.62	4.11	3.38	5.66	4.82	4.80	4.30	3.39
SD (U/mL)		0.44	0.28	0.19	0.25	0.36	0.40	0.15	0.25	0.39	0.31
Bias (U/mL)		0.17	−0.18	0.34	−0.11	−0.09	0.04	0.28	0.52	0.08	−0.08
%TE		18.68	16.48	17.01	14.22	23.23	15.00	12.49	23.88	20.43	20.12
%TE ≤ 20		Pass	Pass	Pass	Pass	Fail	Pass	Pass	Fail	Fail	Fail

**Table 3:** Evaluation of the effect of endogenous substances on the measurement of AQP4-IgG titers.

Endogenous Substances	Concentration	% Difference from Control (95% CI)		
		AQP4-IgG Titers of Samples		
		Low	Middle	High
Hemoglobin	1089 mg/dL	−1.8% (−5.5, 1.8)	3.9% (−1.6, 9.3)	−0.7% (−5.8, 4.4)
Free bilirubin	46 mg/dL	−0.6% (−7.2, 6.0)	0.9% (−2.8, 4.6)	−0.9% (−6.7, 4.9)
Conjugated bilirubin	45 mg/dL	4.1% (−0.9, 9.2)	2.1% (−1.0, 5.3)	5.6% (0.0, 11.3)
Chyle material	1510 FTU	1.9% (−4.1, 7.8)	−2.8% (−8.9, 3.3)	−0.7% (−5.8, 4.4)
Rheumatoid factor	500 IU/mL	3.6% (−1.4, 8.6)	0.0% (−3.6, 3.6)	−0.5% (−3.7, 2.7)
Triglyceride	2000 mg/dL	0.0% (−4.8, 4.8)	0.4% (−4.1, 4.8)	−1.0% (−4.1, 2.0)

**Table 4:** AQP4-IgG titers of AQP4-CBA and AQP4-CLEIA in 33 patients with AQP4-IgG-positive NMOSD. Patients with a measured value of ≥500.0 U/mL were denoted as ≥500.0 U/mL.

Sample No.	AQP4-CBA	AQP4-CLEIA	Sample No.	AQP4-CBA	AQP4-CLEIA
	(Titers)	(U/mL)		(Titers)	(U/mL)
1	16	12.9	18	4096	≥500.0
2	64	4.8	19	8192	≥500.0
3	256	222.0	20	8192	≥500.0
4	256	136.3	21	8192	≥500.0
5	256	79.6	22	16,384	≥500.0
6	256	67.4	23	16,384	≥500.0
7	512	77.5	24	16,384	≥500.0
8	512	27.0	25	32,768	≥500.0
9	512	42.5	26	32,768	≥500.0
10	1024	35.6	27	32,768	≥500.0
11	1024	284.0	28	65,536	≥500.0
12	1024	72.3	29	65,536	≥500.0
13	1024	29.3	30	65,536	≥500.0
14	2048	438.6	31	65,536	≥500.0
15	4096	338.1	32	131,072	≥500.0
16	4096	268.1	33	131,072	≥500.0
17	4096	≥500.0			

NMOSD, neuromyelitis optica spectrum disorder.

**Table 5:** Comparisons of features and performance between assays used for AQP4-IgG measurements.

Principle of Measurement	AQP4-CBA (Microscopic Live CBA) *^1^	AQP4-CLEIA	AQP4-ELISA
Trade name	No commercial kit is available	Under consideration	RSR AQP4 Autoantibody ELISA Version 2 Kit
Manufacture	No commercial kit is available	Medical & Biological Laboratories Co., Ltd.	RSR Limited
Antigen	AQP4-M23 stably expressed HEK-293 cell lines *^2^	Purified AQP4-M23	Purified AQP4-M23
Detection	Fluorochrome-labeled anti-human IgG pAb *^2^	ALP-labeled anti-human IgG pAb	Biotinylated AQP4 and streptavidin peroxidase
Operation	Hand method	Fully automated	Hand method
Measurement time	Observer-dependent	20 min	3 h
Sample volume	Observer-dependent	10 μL	50 μL
Cutoff value	Observer-dependent	10.0 U/mL	3.0 U/mL
Measurement range	Observer-dependent	4.6–500.0 U/mL	1.5–80 U/mL, 1.5–40 U/mL *^3^
Sensitivity	92.0–100.0% [5,20]	97.0%	60.0–83.3% [5,9,20]
Specificity	94.5–100.0% [5,20]	100.0%	96.9–100% [5,9,20]

*^1^ In this table, AQP4-CBA exclusively refers to a microscopic live CBA. *^2^ Example of a microscopic live CBA; each assay depends on observers. *^3^ Measurement range for in vitro diagnostic products in Japan.

## Data Availability

Raw data are available from the corresponding author upon request.

## Abbreviations

The following abbreviations are used in this manuscript:

ALP	Alkaline phosphatase
AQP4-IgG	Aquaporin-4 immunoglobulin G antibodies
CBA	Cell-based assay
CI	Confidence interval
CLEIA	Chemiluminescent enzyme immunoassay
CLSI	Clinical and Laboratory Standards Institute
CNS	Central nervous system
ELISA	Enzyme-linked immunosorbent assay
LoQ	Limit of quantification
MOGAD	Myelin oligodendrocyte glycoprotein antibody-associated disease
MS	Multiple sclerosis
NMOSD	Neuromyelitis optica spectrum disorder
ROC	Receiver operating characteristic
SDS-PAGE	Sodium dodecyl sulfate–polyacrylamide electrophoresis
TE	Total error
